# Polystyrene nanoparticles induce DNA damage and apoptosis in HeLa cells

**DOI:** 10.1016/j.heliyon.2024.e41298

**Published:** 2024-12-18

**Authors:** Antonia Feola, Manoj Madheswaran, Grazia Romano, Awet Ghebretinsae Tewelde, Eunice Wairimu Maina, Gianluca D'Abrosca, Maria della Valle, Mariacristina Cocca, Maria Emanuela Errico, Carla Isernia, Roberto Fattorusso, MariaTeresa Gentile, Gaetano Malgieri

**Affiliations:** a Department of Biology, University of Naples “Federico II” Naples, Italy; b Department of Environmental, Biological and Pharmaceutical Sciences and Technologies, University of Campania “Luigi Vanvitelli”, 81100, Caserta, Italy; c Department of Clinical and Experimental Medicine, University of Foggia, 71122, Foggia, Italy; d Institute of Crystallography–CNR, Via Vivaldi, 43, 81100, Caserta, Italy; e Institute for Polymers, Composites and Biomaterials—CNR, Via Campi Flegrei, 34, 80078, Pozzuoli, Naples, Italy

**Keywords:** Polystyrene, Nanoplastics, Apoptosis, Genotoxicity, HeLa cells, H2AX

## Abstract

Nanoplastics (NPs) are plastic particles, typically less than 100 nm in size, that result from daily life products as well as the degradation of larger plastic debris. Due to their small size and chemical composition, they can interact with biological systems in ways that larger plastic particles cannot. Humans are continuously exposed to NPs and several studies showed the potentially toxic effects of these latter on health. Polystyrene nanoplastics (PS-NPs) are the prevalent form of nanoparticles found in the environment and their cellular uptake can cause cytotoxicity and structural alteration of biomolecules. Thus, there is an urgent need for evaluation of the genotoxic effects of PS-NPs on human cell models. Through different and complementary experimental approaches, we investigated the potential genotoxic and cytotoxic effects of PS-NPs exposure on HeLa cell lines. We highlighted the genotoxic effects of polystyrene nanoplastics by showing the formation of multinuclei and micronuclei in all the studied concentrations and time points, also at short incubation time (6 h) and low concentration. At higher concentrations, we demonstrate the presence of apoptotic and necrotic cells outlining the acute cytotoxic effects of nanoplastics. The genotoxic potential is further highlighted by the presence of low molecular weight DNA fragments in PS-NPs treated cells, and by the relationship between polystyrene nanoplastics and γ-H2AX. Thus, our data provide important insights at a cellular level into the possible risks produced by these nanoparticles and recommend further deeper research studies to address the impacts of nanoplastics on human health.

## 1 Introduction

Plastic is spreading at worrying rates [1] in all the compartments of our environment [2]. The different conditions found in the various environments favor physicochemical phenomena that lead to plastics degradation [3,4] and to the formation of plastic micro- and nano-particles. These particles are classified based on their size as microplastics (MPs <5000 μm) or nanoplastics (NPs <100 nm) [5]. Humans are continuously exposed to MPs and NPs mainly through ingestion, inhalation and skin deposition of voluntarily produced plastic and particles produced by environmental decomposition of the plastic waste [[6], [7], [8], [9]]. Prolonged exposure to these particles is raising considerable concerns in the scientific community regarding the possible risks to human health [10] establishing the urge for further investigations [11,12]. On this issue, growing concerns are posed by NPs compared to MPs, because their smallest dimensions suggest they can be potentially more toxic [13], more reactive, and capable of penetrating living cells [14] In fact, NPs have the potential to interact with cells [15] due to their strong affinity to lipid bilayers [16,17].

The cellular uptake of PS-NPs can cause cytotoxicity [18,19], and structural alterations that affect metabolic processes of cell viability [20]. Moreover, exposure to PS-NPs can lead to genotoxicity, which can occur either through direct interaction with DNA, or from indirect mechanisms such as the generation of reactive oxygen species (ROS) that cause DNA damage [8]. Exposure to PS-NPs has clearly resulted in genotoxicity in certain cell types [[21], [22], [23], [24]]. In mammalian cell cultures, such exposure resulted in the generation of DNA strand breaks and micronuclei [8]. On one hand, recent studies have shown that PS-PNPs can cause both primary DNA and chromosomal damage [25,26]. On the other hand, other studies indicated that PS-NPs exposure did not cause DNA damage in human intestinal epithelial Caco-2 cells [27] and other human cell models [28]. Additionally, the evident cellular uptake of PS-NPs by HeLa cells drastically impacted cytotoxicity and resulted in abnormal gene expression [29]. Thus, there is still a need for further assessment and evaluation of the genotoxic effects of PS-NPs on HeLa cells.

The cytokinesis-block micronucleus (CBMN) assay is an efficient and reliable method to evaluate cytotoxic and genotoxic features of nanomaterials in *in-vitro* cell line studies [30,31]**.** Therefore, the present study aims to investigate the potential genotoxic and cytotoxic effects of PS-NPs exposure on human HeLa cell lines by means of the CBMN assay to evaluate genetic damage, such as micronucleus formation, after exposure to different concentrations of PS-NPs. To assess the viability of cells at different concentrations of PS-NPs and their impact on the cell cycle, we performed flow cytometric analysis for the apoptosis test. We investigated the potential presence of double-strand breaks in DNA evaluating the phosphorylation of the γ-H2AX protein. Our results integrate the limited existing literature providing valuable insights into the possible genotoxic effects of PS-NPs on HeLa cell lines.

## 2 Materials and methods

### 2.1 PS-NPs characterization

1 % solids PS-NPs were bought from Thermo Fisher Scientific – particle technology, CA, USA, (Catalog # 3020A, lot # 273137). The certified mean diameter of PS-NPs was 22 ± 2 nm. Spheres have a density of 1.05 g/cm^3^ and an index of refraction of 1.59 at 589 nm (25 °C) as indicated in the certificates available at www.thermofisher.com. Before the analysis, PS-NPs were sonicated using a Sonics Vibracell (Newtown, CT, USA) ultrasonic processor (500 W, 20 kHz) at an amplitude of 25 % for 10 min to promote the NP dispersion, as previously described [20].

Previous studies demonstrated that PS-NPs particles do not aggregate after suspension in a 10 % FBS-DMEM culture medium [32].

### 2.2 Cell culture

HeLa 229 cell line was obtained from American Type Culture Collection (ATCC, Rockville, MD, USA) and cultured in Roswell Park Memorial Institute (RPMI 1640) medium supplied with 10 % fetal bovine serum (FBS), 1 % pen/strep, and 1 % L-Glutamine (200 mM). Cells were maintained in a constant temperature cell incubator with 5 % CO2 at 37 °C. HeLa cells were treated with 100pM, 100 nM, and 100 μM of nanoplastics and incubated at 37 °C for 24 h. Following incubation, each well was imaged directly under an inverted phase contrast microscope (Eclipse TE300, Nikon) at 10 × magnification.

### 2.3 Cytokinesis-block micronucleus assay

1x10^5^ HeLa cells were plated in a 24-multiwell plate onto poly-L-lysine coated glass coverslips per well, treated with 100pM, 100 nM, and 100 μM of nanoplastics, and incubated at 37 °C for 6 and 24 h as previously described [20]. Cytochalasin B was added to a final concentration of 3 μg/ml and incubated at 37 °C for 24h, then the cells were fixed for 10min in ice-cold 90 % methanol and stained with 20 % Giemsa for 12min. Slides were washed twice with PBS and then allowed to air-dry. Coverslips were mounted with Mowiol and left for several hours to allow the glue to set then were observed and imaged directly under an inverted phase contrast microscope (Eclipse TE300, Nikon) at 10X magnification. The number of multinuclei-positive cells was determined with the AmScope 3.7 software. Data are shown as number of MN-positive cells per field, five fields were captured for each well.

### 2.4 Flow cytometric analysis of apoptosis

For all experiments, 5x10^4^ cells were seeded in 12-well plates into each well using RPMI 1640 complemented as above in a final volume of 500 μl. Cells were cultured at least for 24h to achieve a state of complete adherent before being carried out for experiments, then were treated with PS-NPs (100pM, 100 nM, 100 μM) for 6h, 24h, and 48h. After the times of treatments, HeLa cells were collected by trypsinization and resuspended in 1 ml Phosphate buffered saline 1X (PBS). Apoptotic cells were detected by annexin V-FITC/PI staining assay following the manufacturer procedure (A432; Technologies, Inc.). After, the cells were washed twice with PBS 1X, and resuspended in 100 μl Annexin V binding buffer with 5 μl Annexin-V FITC and 5 μl propidium iodide. Then cells were incubated for 10–15 min in the dark at room temperature and then analyzed by flow cytometry (BD Accuri C6, Becton Dickinson, San Jose, CA).

### 2.5 DNA fragmentation analysis for detection of apoptotic cells

DNA fragmentation is used to detect apoptotic cells. HeLa cells were fixed in ethanol 70 % on ice for 2 h (1 × 10^6^ cells into 10 ml). They were subjected to mild extraction of low-molecular-weight DNA, that leaks from the cells, with 50 μl of phosphate-citrate buffer (0.2M phosphate-citrate buffer, pH 7.8) at 37 °C for 30’. Samples were then subjected to electrophoretic analysis.

### 2.6 Protein analysis

Cytosolic proteins were isolated as previously described [20]. Briefly, proteins were isolated in lysis buﬀer (25 mM Tris-HCl, pH 7.4, 1 mM EDTA, 1 mM EGTA, 0.1 mM NaF, 0.1 mM Na3VO4) and concentration was measured. Western blotting analysis was performed separating 20 μg of proteins on a 10 % SDS-PAGE and electroblotted on PVDF. Membranes were blocked in 5 % milk, and incubated overnight at 4 °C with anti- γH2AX antibody (1:300; Millipore). After incubation with peroxidase-conjugated anti-mouse antibodies (1:5000; Amersham-Biosciences), proteins were revealed by an ECL kit (Millipore). To normalize the protein expression versus loading, membranes were incubated with an anti-β-Actin antibody (1:1000 in TBS-T; Cell Signaling) and revealed as above. Band intensity was quantiﬁed by Image Lab Software ChemiDoc (BioRad).

### 2.7 Statical analysis

All data are presented as means ± S.D. Data were analyzed by one-way and 2-way ANOVA followed by the Bonferroni post-hoc test. Data were analyzed using GraphPad Prism 8.0 (GraphPad Software Inc., La Jolla, CA, USA) with p ≤ 0.05 as the cut-off for statistical significance between groups or the JMP Statistical DiscoveryTM software 6.03 by SAS (Statistical Analysis Software) and tested for normal distribution of variables using the Shapiro-Wilks test (“normal distribution fit” tool—JMP software). Asterisks indicate significance at ∗ p < 0.05, ∗∗p < 0.01, and ∗∗∗p < 0.001 throughout.

## 3 Results and discussion

### 3.1 PS-NPs induce micronuclei formation in HeLa cells

**Fig. 1:**
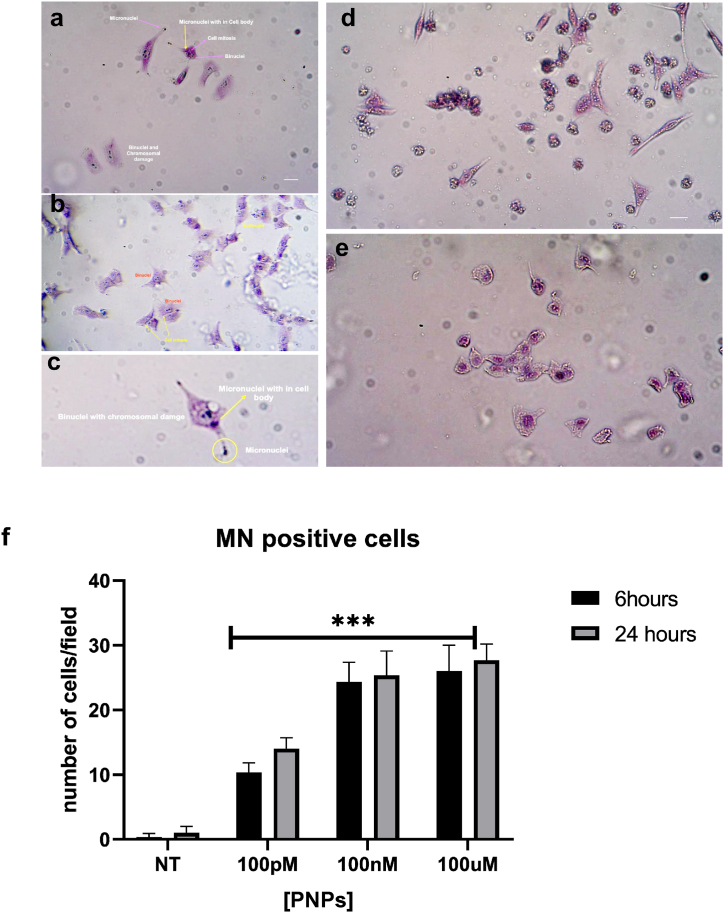
*Cytokinesis-Block Micronucleus Assay* on HeLa Cells after exposure to PS-NPs: PS-NPs induce micronuclei formation in HeLa cells treated with 100 nM **(a)** and 100 μM **(b)** soon after 6h of incubation. **(c)** Image detail of micronuclei visible both in the nucleus and the cytoplasm. PS-NPs incubation time (24h):100 nM **(d)** and 100 μM **(e)**, Images are representative of three independent experimental settings. **(f)** MN formation at 6h and 24h of PS-NPs incubation**.** n = 18 for each experimental condition. Data are expressed as mean ± S.D. ∗∗∗p < 0.0001.

Micronuclei (MN) are small nucleus-like structures formed from chromosome fragments or whole chromosomes that lay behind during anaphase and fail to integrate into daughter nuclei. This process can be indicative of genomic instability and is commonly used as a biomarker for genotoxicity and chromosomal damage [33]. Exposure to chemicals, radiation, and other genotoxic agents can damage DNA, leading to chromosome breakage or missegregation. The correlation between nanoplastics and micronuclei formation is an emerging area of research, directed towards uncovering the potential genotoxic effects of nanoplastic particles. We investigated whether PS-NPs induce micronucleus formation in a human cell line (HeLa) using the CBMN assay. We opted for two different incubation periods, 6 h and 24 h, to capture both short-term and longer-term effects on cellular morphology and genetic stability. After incubating nanoplastics for 6 h, numerous viable cells were still observed, although some exhibited signs of damage, including the formation of multinuclei (Fig. 1a–c).

HeLa cells treated with 100 nM (Fig. 1a) and 100 μM (Fig. 1b) soon after 6h of incubation showed micronuclei both in the nucleus and the cytoplasm; cells were still alive, and their morphology was saved even if micronuclei were visible in a large part of them. At 100 nM of PS-NPs, cell necrosis, characterized by cellular death and breakdown, was observed in response to 24-h exposure highlighting PS-NPs potential cytotoxic effects (Fig. 1d). PS-NPs treatment at extended incubation periods resulted in severe cell death, indicating the potent cytotoxic effects of prolonged exposure also to the highest nanoplastic levels (100 μM) (Fig. 1e). Upon examination, we consistently observed the formation of multinuclei and micronuclei across all concentrations and time intervals. It's noteworthy that these formations manifest rapidly after exposure to PS-NPs, even at lower doses, suggesting a high genotoxic potential and effects independent of dose and time.

### 3.2 Short PS-NPs treatment times causes apoptosis in HeLa cells

Once generated, MNs can exist for several cellular generations [34] since they are protected by an atypical nuclear envelope and, can acquire aberrant epigenetic chromatin marks that may persist for future cellular generations. Furthermore, the MN nuclear envelope can rupture, leading to the accumulation of MN DNA damage and subsequent chromosomal recombination (chromothripsis), as well as cell death.

**Fig. 2:**
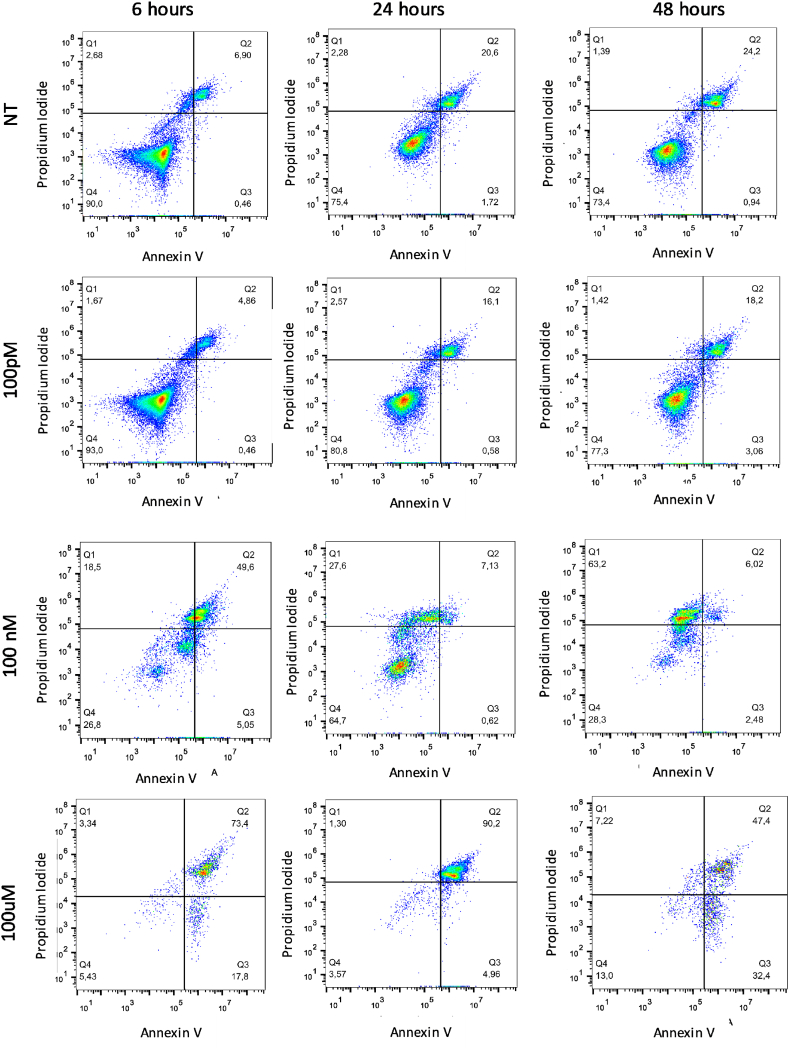
Apoptosis analysis by Cytofluorimetric Annexin V-FITC/PI assay: HeLa cells treated with PS-NPs at the indicated doses in different time points. Staining with Annexin V or PI resulted in matching fractions of vital cells, early/late apoptosis, and necrotic cells. Cells in region Q4 represent living cells, cells in Q3 early apoptotic cells, cell in Q2 late apoptotic cells, and cells in Q1 those with a damaged membrane only. Each panel corresponds to a representative analysis of at least three separate experiments.

**Fig. 3:**
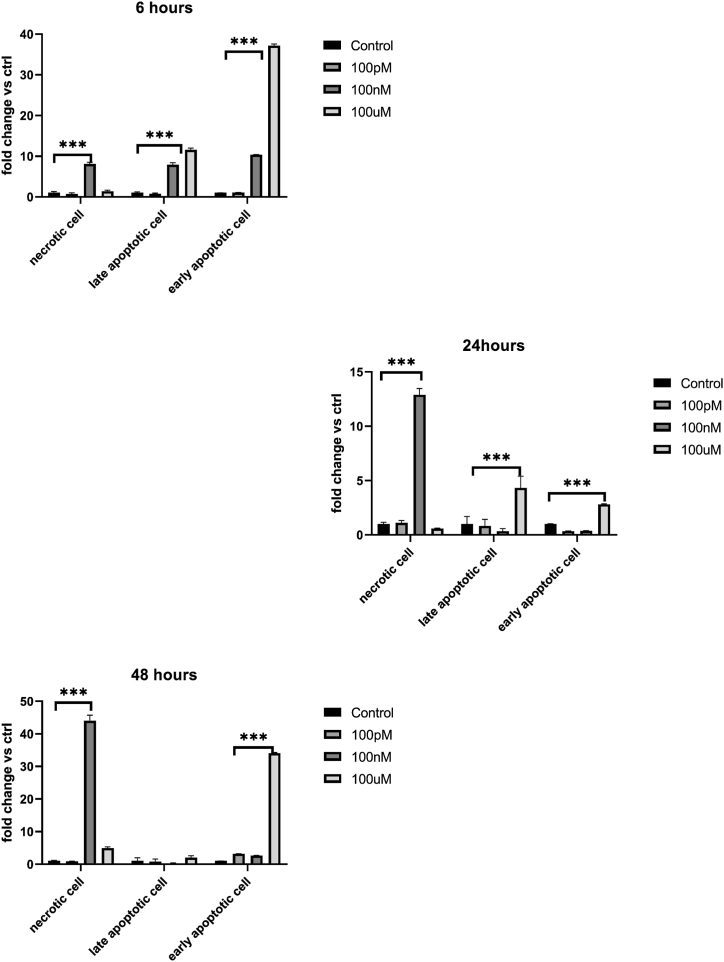
**Schematic representation of cell death analysis**: Graphic representation of HeLa cells treated with PS-NPs at the indicated doses in different time points, stained with Annexin V and PI, and analyzed with BDAccuriC6 Cytofluorimeter. Histograms represent the mean of each treatment compared to untreated control. Results in fractions are reported: necrotic cells, late apoptotic cell, and early apoptotic cell. The statistical analysis derives from at least three experiments in triplicate (n ≥ 9; mean ± SD); ∗p < 0.05, ∗∗p < 0.01, and ∗∗∗p < 0.001 compared to untreated cells.

**Fig. 4:**
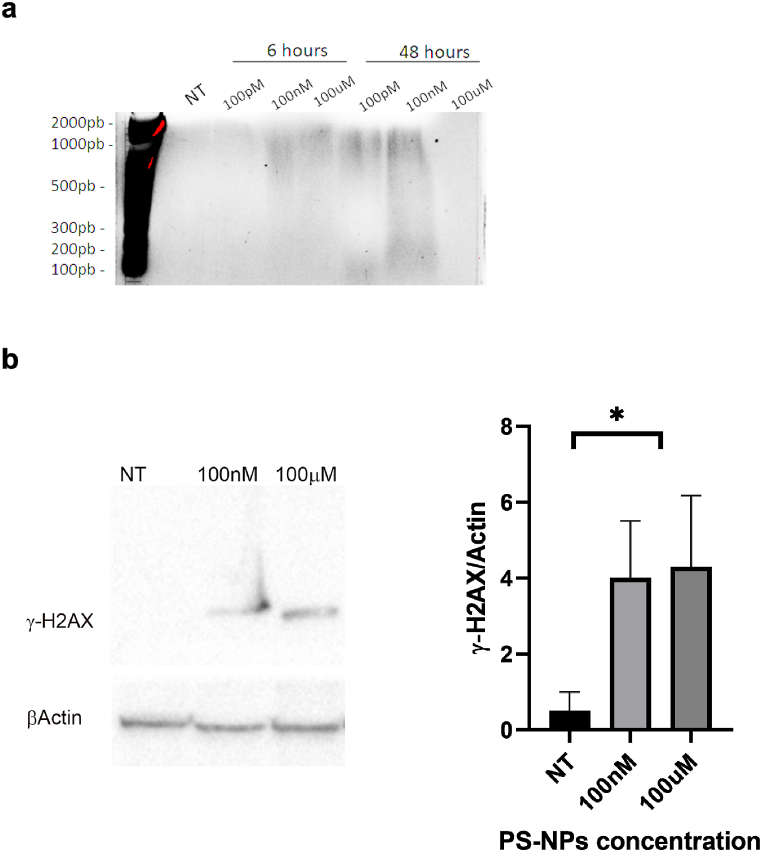
**Western blotting analysis**. (**a)** DNA fragmentation in low molecular weight fragments is visible in extracts from PS-NPs treated cells after 6 and 48 h of treatment at 100 nM, and 100 μM of concentration. The image is representative of three independent experiments. **(b**) Western blotting analysis of protein extract from HeLa cells treated or not (NT) with increasing doses of PS-NPs as indicated. γ-H2AX positivity indicates that PS-NPs treatment induces double-strand break in DNA from treated cells but not in untreated cells (NT). No significant difference in band intensity is detected, as shown in the graph. Data are represented as mean ± SEM of at least 3 experiments, ∗p < 0,05. Data are representative of three independent experiments.

To further elucidate the possible mechanism behind PS-NPs -induced cell death, we performed a cytofluorimetric measurement following the Annex V/PI staining. During apoptosis, the phosphatidyl serine (PS) in the inner face of the cell membrane flips out the membrane [35]. Annexin V detected PS but alone cannot differentiate between apoptotic and necrotic cells, so we stained cells with PI too. In this case, the necrotic cells could be differentiated from the apoptotic cells, because the early apoptotic cells exclude PI, while late-stage apoptotic cells and necrotic cells were stained positively. To this end, HeLa cells were treated with increasing doses of PS-NPs (100pM, 100 nM and 100 μM) for 6h, 24h and 48h. The results obtained demonstrated that 100 nM and 100 μM are highly toxic doses for HeLa cells (Fig. 2). Interestingly, after only 6h of treatment, more than 50 % ± 0,51 of cells appeared double positive, indicating a late stage of apoptosis. This rapid onset of apoptosis suggests that PS-NPs quickly trigger cell death pathways, particularly at higher doses. This condition gets worse at the highest dose (73,3 % ± 0,43) indicating that higher concentrations of PS-NPs exacerbate cellular damage, leading to quicker progression to late-stage apoptosis (Table 1). On the other hand, after 48 h of treatment, at 100 nM about 60 % (66,6 % ± 1,72 Vs 1,52 ± 0,16 of CTR) was necrotic, while following 100 μM treatment HeLa cells underwent death through the apoptotic process soon after 6h (see Table 1). Specifically, the most of cells (73,35 % ± 0,43) went to late-stage apoptosis, while only 18,03 % ± 0,42 were still in the early stage. Those cells that survived until 48h later stimulation, also died due to apoptosis as demonstrated by the single positivity to Annexin V (32.7 % ± 0,25) at the highest dose (Fig. 2, Table 1). If compared to the untreated control, in cells treated for 6h with 100 nM PS-NPs significant increase (8–10 fold) (p < 0.001) the number of cells which went to cell death, indifferently from the molecular mechanism. It is interesting to note that, increasing the exposure time of 100 nM PS-NPs to 24 and 48h, significantly increased the fold chance of necrotic cells compared to control, at 12 and 44 fold, respectively. Indeed, the highest dose (100 μM) caused a 37-fold increase in cells dying by early apoptosis after 24h (Fig. 3). To corroborate these data, mild extraction of low-molecular-weight DNA was performed, and the results obtained demonstrate a DNA fragmentation between 100 nM e 100 μM already after 6h (1000-2000bp), which is dose-dependent and becomes worse over time, in particular, after 48 h of PS-NPs exposure molecular weights below 500bp are observed in DNA (Fig. 4a). This indicates that 100 μM of PS-NPs cause rapid and severe cellular stress, pushing cells quickly through the apoptotic process due to the rapid and potent genotoxic and cytotoxic effects of PS-NPs. The quick progression to late-stage apoptosis suggests a strong immediate impact on cellular integrity and function. The transition from apoptosis at higher doses and short exposure times to necrosis at moderate doses and longer exposure times highlights the dynamic cellular response to PS-NPs. It suggests that initial exposure might trigger programmed cell death (apoptosis), but prolonged stress and damage can lead to necrotic cell death due to the failure of cellular repair mechanisms. Although the mechanisms and morphologies of apoptosis and necrosis differ, there is an overlap between these two processes. Evidence indicates that necrosis and apoptosis represent morphologic expressions of a shared biochemical network described as the “apoptosis-necrosis continuum” [36] two factors that will convert an ongoing apoptotic process into a necrotic process with a decrease in the availability of caspases and intracellular ATP [37,38]. Sun et al. demonstrated that PS-NPs exposure induces mitochondrial and ATP impairment in Leydig cells [39] and insufficient ATP levels impair cellular functions and eventually lead to cell death through necrosis and can induce apoptosis via both intrinsic and extrinsic pathways. The observed effects are both concentration and time-dependent, emphasizing the importance of dose and exposure duration in evaluating the toxicological impact of PS-NPs. Higher doses lead to rapid apoptosis, while prolonged exposure at lower doses results in delayed apoptosis or necrosis. Altogether, our experiments highlight a sort of threshold concentration of PS-NPs above which cellular repair mechanisms are overwhelmed, leading to irreversible damage and cell death. Indeed, even at shorter incubation periods, necrotic cells were observed at higher concentrations, underscoring the acute cytotoxicity of nanoplastics, particularly at elevated doses.

**Table 1:** The table reports the mean of at least 3 experiments expressed as the percentage of the cells that show positive signals for annex V and/or PI compared to the total cells ±standard deviation indicated in Fig. 4 of all quadrants. Cells in region Q4 represent living cells, cells in Q3 early apoptotic cells, cell in Q2 late apoptotic cells, and cells in Q1 those with a damaged membrane only.

Q1	6h	24h	48h
**NT**	2,37 % ± 0,32	2,21 % ± 0,15	1,52 % ± 0,16
**100pM**	1,66 % ± 0,29	2,44 % ± 0,22	1,32 % ± 0,8
**100 nM**	18.8 % ± 0,45	28,3 % ± 0,57	66,6 % ± 1,72
**100uM**	3,23 % ± 0,27	1,27 % ± 0,3	7,44 % ± 0,36
**Q2**	**6h**	**24h**	**48h**

**NT**	6,35 % ± 0,25	20,72 % ± 0,69	23,89 % ± 0,97
**100pM**	4,83 % ± 0,20	17,04 % ± 0,60	19 % ± 0,80
**100 nM**	50 % ± 0,51	7,15 % ± 0,22	6,07 % ± 0,12
**100uM**	73,35 % ± 0,43	89,30 % ± 1,08	48,05 % ± 0,57
**Q3**	**6h**	**24h**	**48h**

**NT**	0,48 % ± 2	1,77 % ± 3	0,96 % ± 2
**100 pM**	0,50 % ± 4	0,60 % ± 2	3,05 % ± 3
**100 nM**	5,04 % ± 3	0,65 % ± 3	2,54 % ± 7
**100 uM**	18,03 % ± 0,42	4,98 % ± 4	32,7 % ± 0,25

### 3.3 Oxidative effects on HeLa cell due to PS- NPs treatment

All these effects could be due to several direct and indirect mechanisms such as oxidative stress and reactive oxygen species (ROS) production. Indeed, nanoplastics can generate ROS, including superoxide anions, hydrogen peroxide, and hydroxyl radicals, almost immediately upon entering a cellular environment and can cause oxidative damage [40]. Nanoplastics (NPs) can also induce an inflammatory response, leading to the release of pro-inflammatory cytokines such as TNF-α that can activate signaling pathways triggering ROS production rapidly which can exacerbate cellular damage and lead to necrosis [41]. Moreover, NPs can disrupt mitochondrial function, producing mitochondrial ROS (mtROS) and decreased ATP [42]. Studies postulated also other mechanisms behind NPs-induced cell damage. They can also affect cellular calcium channels and pumps, leading to dysregulated calcium levels and eventually cell death [43]. A common feature of all these cellular events is indirect DNA damage: increased ROS production contributes to DNA damage as well as elevated intracellular calcium which can activate nucleases and cause DNA fragmentation resulting in single-strand breaks (SSBs) and double-strand breaks (DSBs). Indeed, NPs can penetrate cellular membranes, enter the nucleus, and directly interact with DNA [8]. Microscopy studies have shown the presence of NPs in the nucleus within hours of exposure, correlating with increased DNA damage markers like γ-H2AX [40]. To corroborate our data, we performed the analysis of the presence of γ-H2AX which represents an early cellular response to the induction of DNA DSBs. Phosphorylation of the Ser-139 residue of the histone variant H2AX induces the formation of γ-H2AX, whose detection has emerged as a highly specific and sensitive molecular marker for monitoring DNA damage initiation and resolution [44]. As shown in (Fig. 4b), exposure to increasing concentrations of PS-NPs results in γ-H2AX formation, confirming DNA damage. The correlation between NPs and γ-H2AX highlights the potential genotoxicity of nanoplastic particles.

By inducing oxidative stress, physical DNA damage, and inflammatory responses, NPs can lead to the formation of γ-H2AX, initiating DNA repair mechanisms, cell cycle arrest, and potentially apoptosis or senescence [45]. Double-strand breaks (DSBs) in DNA are among the most severe forms of genetic damage. The repair of DSBs is crucial for maintaining genomic stability. If not properly repaired, DSBs can lead to mutations, chromosomal rearrangements, and cell death, all of which are implicated in the development of various pathologies that can range from cancer to neurological disorders and immunodeficiencies. Improper repair of DSBs can introduce mutations, including point mutations, insertions, deletions, and larger chromosomal rearrangements, and can activate oncogenes or inactivate tumor suppressor genes, driving cancer progression. DSBs also contribute to genomic instability, a hallmark of cancer. Moreover, cells with high genomic instability have increased rates of mutations and chromosomal changes, leading to cancer heterogeneity and resistance to therapy [46]. Moreover, neurons are particularly sensitive to DNA damage due to their long lifespan and limited capacity for cell division. DSBs in neuronal DNA can impair cellular function and viability, leading to neurodegeneration. Accumulation of DNA damage, including DSBs, is implicated in Alzheimer's and Parkinson's diseases [47]. Moreover, DSBs play a crucial role in V(D)J recombination, a process essential for the development of a diverse repertoire of antibodies and T-cell receptors. Defective repair of DSBs can impair immune function. For example, preliminary studies suggest that severe combined Immunodeficiency (SCID) is caused by PS-NPs induced mutations in genes like RAG1, RAG2, or DNA ligase IV, which are involved in DSB repair during V(D)J recombination [39]. Moreover, PS-NPs could induce DNA damage in endothelial cells and contribute to the development of atherosclerosis, a major cause of cardiovascular diseases and persistent DSBs could trigger chronic inflammation, which is linked to various cardiovascular conditions [48]. PS-NPs induced DSBs in germ cells can lead to infertility and other reproductive issues and above all, DNA damage in germ cells can be passed on to offspring, potentially causing developmental defects and diseases in subsequent generations [49].

## 4 Conclusions

Our study reports the interaction of polystyrene nanoplastics with HeLa cells to provide important insights at a cellular level into the health impact and possible risks produced by these nanoparticles. We highlight the genotoxic effects of PS-NPs by showing the formation of multinuclei and micronuclei in all the studied concentrations and time points. However, after deep analysis of cell death, we observed severe cellular damage and cell death at higher concentrations and longer incubation times, outlining a dose-dependent cytotoxicity. It is worth noting that at shorter incubation time (6 h), our data at higher PS-NPs concentrations demonstrate the presence of apoptotic cells, outlining the acute cytotoxic effects of nanoplastics.

The pro-apoptotic properties of these PS-NPs were confirmed through Annexin V assays that provided clear evidence of induction of apoptosis. The genotoxic potential is further highlighted by the relationship between PS-NPs and γ-H2AX. Oxidative stress, physical DNA damage and inflammatory responses induced by nanoplastics can lead to the formation of γ-H2AX that is associated with initiating DNA repair mechanisms, cell cycle arrest, and potentially apoptosis or senescence. Our data cannot demonstrate that these PS-NPs are able to penetrate the nucleus, however, several evidences demonstrate PS-NPs are able to penetrate the nucleus in a size dependent manner [29,[50], [51], [52]]. Thus, altogether, our data demonstrate that polystyrene nanoparticles already at low doses induce a rapid (only 6 h later) DNA damage in HeLa cells, suggesting a possible direct or indirect interaction of PS-NPs with DNA.

These results recommend more scientific efforts and additional research designed to address the impacts on human health of nanoplastics.

## CRediT authorship contribution statement

**Antonia Feola:** Writing – original draft, Methodology, Investigation. **Manoj Madheswaran:** Methodology, Investigation. **Grazia Romano:** Validation, Methodology, Investigation. **Awet Ghebretinsae Tewelde:** Validation, Methodology. **Eunice Wairimu Maina:** Validation, Methodology. **Gianluca D'Abrosca:** Writing – review & editing, Validation, Supervision. **Maria della Valle:** Validation, Methodology. **Mariacristina Cocca:** Writing – review & editing, Validation. **Maria Emanuela Errico:** Writing – review & editing, Validation. **Carla Isernia:** Validation, Supervision, Resources. **Roberto Fattorusso:** Validation, Supervision, Resources. **MariaTeresa Gentile:** Writing – review & editing, Validation, Supervision, Resources, Conceptualization. **Gaetano Malgieri:** Writing – review & editing, Validation, Supervision, Resources, Funding acquisition, Conceptualization.

## Data availability

Data will be made available on request.

## Declaration of competing interest

The authors declare the following financial interests/personal relationships which may be considered as potential competing interests:Gaetano Malgieri reports financial support was provided by 10.13039/501100009448University of Campania Luigi Vanvitelli. If there are other authors, they declare that they have no known competing financial interests or personal relationships that could have appeared to influence the work reported in this paper.
